# Ubiquitous Sialometabolism Present among Oral Fusobacteria

**DOI:** 10.1371/journal.pone.0099263

**Published:** 2014-06-04

**Authors:** Saori Yoneda, Brandon Loeser, Joseph Feng, John Dmytryk, Fengxia Qi, Justin Merritt

**Affiliations:** 1 Department of Oral Medical Science, Ohu University, Fukushima, Japan; 2 Department of Periodontology, University of Oklahoma Health Sciences Center, Oklahoma City, Oklahoma, United States of America; 3 Department of Microbiology and Immunology, University of Oklahoma Health Sciences Center, Oklahoma City, Oklahoma, United States of America; 4 Division of Oral Biology, University of Oklahoma Health Sciences Center, Oklahoma City, Oklahoma, United States of America; University of Toronto, Canada

## Abstract

*Fusobacterium nucleatum* is a ubiquitous member of the human oral flora and is associated with the development of periodontitis and a variety of other types of polymicrobial infections of the mucosa. In the oral cavity, this species is one of the few that is prevalent in both healthy and diseased subgingival plaque. Using microarray analysis, we examined the transcriptional response of *F. nucleatum* subspecies *nucleatum* to whole blood in order to identify some of the genetic responses that might occur during the transition from health to disease. From these studies, we identified a sialic acid catabolism operon that was induced by the presence of blood. We subsequently confirmed that this operon was inducible by the presence of synthetic sialic acid, but we found no evidence suggesting sialic acid was used as a major carbon source. However, this organism was found to possess a *de novo* synthesized surface sialylation ability that is widely conserved among the various *F. nucleatum* subspecies as well as in *F. periodonticum*. We provide evidence that fusobacterial sialylation does occur in the oral cavity irrespective of health status. Interestingly, only a minority of fusobacterial cells exhibit surface sialylation within dental plaque, whereas most cells are uniformly sialylated when grown in pure culture. The implications of these results are discussed.

## Introduction

Sialic acids are a group of nine-carbon sugars that are the primary terminal residues found on mammalian glycoproteins with the C-5 acetylated sugar (Neu5Ac) being the predominant variant [1]. Given the large number of terminal glycosylations typically found on mammalian proteins, these molecules also form the primary interface between bacteria and host cells. As such, a growing body of evidence implicates sialic acid (Neu5Ac in particular) as a crucial mediator of the host-pathogen interaction, especially at mucosal surfaces [2], [3]. Many mucosal pathogens and members of the human flora possess sialidases that can remove these residues from sialylated glycoproteins, like the mucins [4]. Consequently, sialic acid comprises a readily available carbon and nitrogen source at numerous sites throughout the human body. Sialic acid also performs an essential role in protecting host cells from spurious serum complement activation and is required to engage immune cell sialic acid-binding immunoglobulin-like lectins (siglecs) [5]. Most siglecs inhibit immune cell activation and likely serve important roles in preventing inflammatory mediated tissue destruction [6]. Interestingly, recent studies have shown that these serum resistance and siglec engagement functions of sialic acid can also be exploited by certain mucosal pathogens that have the ability to sialylate their cell surfaces [7]–[15].

Bacterial sialometabolism can be grouped into four general categories: 1) organisms that catabolize sialic acid, 2) organisms that synthesize sialic acid *de novo* for surface sialylation, 3) organisms that both catabolize sialic acid and scavenge exogenous sialic acid for surface sialylation, and 4) organisms that can both catabolize sialic acid and synthesize it *de novo* for surface sialylation [2]. For sialic acid catabolism, the required genes are often arranged in an operon or cluster of *nan* genes, of which *nanA, K,* and *E* encode the core catabolic enzymes that convert Neu5Ac into N-acetylglucosamine 6-phosphate (GlcNAc-6-P) [3]. This metabolite can then feed into a variety of energy-generating or biosynthetic pathways. The *nan* gene loci also frequently encode sialic acid transport systems that are often either ABC transporters, symporters/antiporters, or tripartite ATP-independent periplasmic (TRAP) transporters [3]. For those bacteria that can synthesize sialic acid *de novo*, the necessary genes are often found in an operon or cluster of *neu* genes. The core functions for this pathway are encoded by *neuA, B,* and *C*, which convert UDP-N-acetylglucosamine (UDP-GlcNAc) into N-acetylmannosamine (ManAc) followed by Neu5Ac and finally CMP-Neu5Ac [3]. CMP-Neu5Ac can then serve as a substrate for sialyltransferases that are responsible for modifying bacterial surface molecules. Sialic acid catabolic and synthetic pathways can both be found distributed among organisms inhabiting numerous mucosal sites in the body [16]; however, oral bacteria have surprisingly only been demonstrated to catabolize sialic acid [17]. Indeed, currently available genome sequence data indicate a large number of oral bacteria encode sialidases and/or the aforementioned *nan* genes, whereas *neu* gene orthologs have only been observed in one of the three sequenced subspecies (ssp.) of *Fusobacterium nucleatum* (ssp. *polymorphum*) [17].

Traditionally, *F. nucleatum* has been considered an important species for the formation of human dental plaque and is implicated in the development of gingivitis and periodontal disease [18]. This species is a ubiquitous member of the human oral flora and can be found at both healthy and diseased sites in the oral cavity. Though, its prevalence increases substantially with the onset of periodontitis [18]. Recently, *F. nucleatum* has also been recognized as part of the resident mucosal flora in the human intestine [19]. One of the more unusual features of this organism is its ability to adhere to a vast diversity of other bacterial species [20]. *F. nucleatum* is also strongly adherent and highly invasive for a large variety of human cell types [21]–[25] and has been isolated from more infections outside of the oral cavity than any other oral bacterial species [18]. Furthermore, recent studies of *F. nucleatum* suggest that this organism may also play a prominent role in the pathogenesis of a variety of diseases of the gut, such as appendicitis [26], [27] and inflammatory bowel diseases [19], [28], and can contribute to adverse pregnancy outcomes [29], [30] and possibly colon cancer [31], [32]. However, due to the limited genetic system for this species, little is known about its pathobiology.

Given that *F. nucleatum* exists in association with both healthy and diseased gingiva, we were curious to examine the effect of blood upon the *F. nucleatum* transcriptome. From these studies, we found that the low levels of free sialic acid in blood likely serve as inducers of the *F. nucleatum* ssp. *nucleatum nan* operon. However, unlike other bacteria that possess these genes, sialic acid could not serve as a prominent carbon source, which implicates a limited role for sialic acid in ATP generation. In addition, we were able to detect a surface sialylation ability in this organism using a fluorescent sialic acid staining procedure. By examining a panel of mostly clinical isolates, we further demonstrate that surface sialylation is likely a general ability of the various subspecies of *F. nucleatum* as well as *F. periodonticum*, the other major fusobacterial species in the oral cavity. Thus, surface sialylation appears to be a highly conserved feature of oral fusobacteria, whereas sialic acid catabolism is probably minimal, even in strains possessing the *nan* genes.

## Materials and Methods

### Bacterial strains and culture conditions

Wild-type strains of *Fusobacterium nucleatum* and *Fusobacterium periodonticum* (3 laboratory strains and 14 clinical isolates) used in this study are listed in Table 1. All clinical isolates were identified by comparing the sequences of the 16S rRNA and zinc protease genes as described by Kim *et al.* [33]. All fusobacteria were grown anaerobically (85% N2, 10% CO2, and 5% H2) at 37 °C on Modified CDC Laked Blood Anaerobe medium agar plates (15 g L^−1^ agar, 10 g L^−1^ pancreatic digest of casein, 10 g L^−1^ proteose peptone, 5 g L^−1^ yeast extract, 5 g L^−1^ NaCl, 2 g L^−1^ L-glutamate, 0.4 g L^−1^ L-cysteine, 5% laked sheep blood, 10 mg L^−1^ vitamin K and 5 mg L^−1^ hemin) or in a previously described semi-defined medium (SDM) [34] containing either animal or vegetable derived proteose peptone as a carbon source.

**Table 1:** Bacterial strains used in this study.

Wild-type strain	Species	Source
JM17	*F. nucleatum* ssp. *animalis*	Clinical isolate
JMJK	*F. nucleatum* ssp. *animalis*	Clinical isolate
JMSK1	*F. nucleatum* ssp. *animalis*	Clinical isolate
FQG51A	*F. nucleatum* ssp. *animalis*	Clinical isolate
25586	*F. nucleatum* ssp. *nucleatum*	ATCC; genome reference strain
23726	*F. nucleatum* ssp. *nucleatum*	ATCC; laboratory strain
12230	*F. nucleatum* ssp. *nucleatum*	ATCC; laboratory strain
JMSY2	*F. nucleatum* ssp. *polymorphum*	Clinical isolate
ZZL1	*F. nucleatum* ssp. *polymorphum*	Clinical isolate
FQ1	*F. nucleatum* ssp. *polymorphum*	Clinical isolate
JMP2	*F. nucleatum* ssp. *polymorphum*	Clinical isolate
JMP4	*F. nucleatum* ssp. *polymorphum*	Clinical isolate
JMTC3	*F. nucleatum* ssp. *polymorphum*	Clinical isolate
JMP2A	*F. nucleatum* ssp. *vincentii* *	Clinical isolate
JMGN1	*F. nucleatum* (unnamed ssp.)	Clinical isolate
JMSK2	*F. periodonticum*	Clinical isolate
SYJL4	*F. periodonticum*	Clinical isolate

*F. nucleatum ssp. *vincentii* and ssp. *fusiforme* have been proposed to comprise a single subspecies [33].

### RNA extraction

To obtain RNA samples for microarray and RT-PCR, cells were first grown to stationary phase in 1 ml SDM. Stationary phase cultures were then diluted 1∶200 in 20 ml SDM and grown to log phase (OD_600_ 0.3–0.5) before adding either a final concentration of 33% whole sheep blood (Lakewood Biochemical) or synthetic N-acetylneuraminic acid (0.02 mM, 0.2 mM, and 2 mM) (Calbiochem). Samples were further incubated anaerobically for 3 hr at 37 °C. After the assay period, the cells were immediately centrifuged at 4 °C in a prechilled refrigerated centrifuge. RNA was extracted from cell pellets using a slightly modified version of a previously described method [34]. Briefly, the cell pellets were resuspended in 500 µl chilled Tris-EDTA buffer on ice and transferred to a 2 ml screw-cap tube containing 500 µl 0.1-mm silica beads (Biospec), 900 µl of 70 °C preheated acidic phenol (pH 4.3) and 140 µl of 10% (wt/vol) sodium dodecyl sulfate. Tubes were then submitted to two consecutive 30 sec homogenization cycles with a FastPrep-24 system (MP Biomedicals) set at a speed of 6.0 M s^−1^. This was followed by acidic phenol-chloroform extraction and isopropyl alcohol precipitation. After treatment with RNase-free DNase (Promega), RNA was further purified over RNeasy spin columns (Qiagen). The resulting RNA integrity was confirmed by the presence of clearly defined rRNA bands in agarose gels and quantified by measuring UV absorbance at 260 nm.

### Microarray and qRT-PCR

Microarray and qRT-PCR were performed as described previously [34]. The protocol is briefly summarized as follows. For microarray, 15 µg of total RNA was used for cDNA synthesis with SuperScript II reverse transcriptase (Invitrogen) and fragmented with DNase I (Roche). Hybridization, washing, and scanning of the custom GeneChip expression microarrays (Affymetrix) were performed according to the protocol provided by Affymetrix. Microarray data processing and analysis employed the GeneChip operating software (GCOS) version 1.4. Probe intensity values, gene expression changes, and statistical analyses are calculated automatically in GCOS using a proprietary automated script provided by Affymetrix. The significance threshold for gene expression data was p≤0.01 with a differential expression cutoff value of ≥2-fold. Microarray data have been deposited in the NIH Gene Expression Omnibus database under the accession number GSE36410.

For qRT-PCR, 300 ng total RNA was used for cDNA synthesis using AffinityScript Multiple Temperature Reverse Transcriptase (Agilent Technology) according to the manufacturer's protocol. qPCR was performed with PerfeCTa SYBR Green SuperMix with ROX (Quanta), and the DNA gyrase gene *gyrA* was used as the housekeeping gene reference. All samples included a no-RT control to assess genomic DNA contamination in the reaction. Primers used for qPCR are listed in Table 2. An unpaired Student's *t*-test was used to assess the significance of gene expression changes within the *nan* operon in response to synthetic sialic acid.

**Table 2:** qPCR primers used in this study.

Primer name	Sequence (5′→3′)	Target
Fnuc gyr F	TTAAATGGAGCAATAGGAATAGCTGT	*gyrA;* FN2125
Fnuc gyr R	TCCGTCAACCAATTCTCCTAAATT	*gyrA;* FN2125
Fn0114 RT F	GACAGAAGGAGCTTTTAATCCAGAAT	FN0114
Fn0114 RT R	CATTGCTGGTCTGATAACTTTACCTTT	FN0114
Fn0664 RT F	AAATGGCAAGCTGCAGGAA	FN0664
Fn0664 RT R	TTGATGCCCACCACTTTTTG	FN0664
Fn1273 RT F	AAATGGCAGCTGTCGGAGAT	FN1273
Fn1273 RT R	CTTGAACACCACCTGTCCATTC	FN1273
Fn1472 RT F	TCCAGGAGCAGCTGCAAACT	FN1472
Fn1472 RT R	GCCATAGGTGTAGGTGCTGCTT	FN1472
Fn1893 RT F	ATGGAATGGCTTATGTTCATGAAA	FN1893
Fn1893 RT R	TCCTACCTTGGCTTGTATTTGTTCT	FN1893
Fn1941 RT F	GGTGCACCCCCAGGATATG	*clpB*; FN1941
Fn1941 RT R	TACATTAAATACATCAGGATGAGCCTTT	*clpB*; FN1941

### Sialic acid staining assay

Stationary phase cultures were diluted 1∶200 in 4 ml SDM and grown to log phase (OD_600_ 0.3–0.5). For the analysis of clinical subgingival plaque samples, plaques were dispersed by brief sonication and adjusted to OD_600_ 0.3. Cells were then washed twice with phosphate buffered saline (pH 7.4) and stained using an aniline catalyzed periodate oxidation protocol as described by Zeng *et al.* [35]. This process selectively introduces an aldehyde group at the C7 position of sialic acid that can be subsequently conjugated with biotin and labeled with streptavidin dyes. The protocol is briefly summarized. First, cells were suspended in PBS containing 1 mM sodium periodate (Sigma) and treated for 30 min at 4°C. The cells were then quenched with 1 mM glycerol before washing in PBS. Next, periodate treated cells were suspended in 1 ml PBS containing 100 µM aminooxy biotin (Invitrogen) and 10 mM aniline (Sigma). The reaction was performed at 4°C for 90 min with gentle agitation on a rocking tray. The cells were washed with PBS and stained with 3.2 µg DyLight 594 conjugated streptavidin (Jackson Immunoresearch Laboratories) in 500 µl PBS for 1 hr on ice, followed by washing with PBS twice. Finally, cells were resuspended in 300 µl PBS and visualized by phase contrast and epifluorescence microscopy using an Olympus BX61 fluorescence microscope with image capture. All images were captured using a 100x oil immersion lens and Spot camera software v3.5. Fluorescence intensity values were determined using ImageJ image analysis software (NIH). For each stained sample, average fluorescence was determined by analyzing the fluorescence intensity values of 10 randomly selected fluorescent cells from 2 separately acquired images (20 cells total) and then averaging all of the values. In order to directly compare fluorescence values between strains, all labeling experiments included sheep erythrocytes stained in parallel with the bacteria followed by image analysis using the same protocol. Final fluorescence values for each fusobacterial isolate were then expressed as a ratio relative to erythrocyte fluorescence. To calculate the percentage of fluorescent cells, 10 randomly selected cells were chose from 2 separately acquired phase contrast images (20 cells total) and compared with the corresponding fluorescent images to determine if the selected cells exhibited fluorescence. Percentages were calculated based upon the number of fluorescent cells/20.

### Treatment with neuraminidase

To remove terminal sialic acid residues from fusobacteria, cells were incubated in citrate buffer (pH 6.0) with 1.25 U of Clostridium perfringens neuraminidase type V (Sigma) for 16 hr at 37°C. Samples were then fluorescently labeled using the previously described periodate oxidation protocol. Each sample included a neuraminidase-free positive control that exhibited fluorescence after surface labeling.

### Subgingival plaque collection

A total of 20 subgingival plaque samples (10 health and 10 disease) were collected from the University of Oklahoma Health Sciences Center dental clinic as part of routine treatment procedures and analyzed anonymously. The collection protocol was previously reviewed by the University of Oklahoma Health Sciences Center Institutional Review Board and granted approval from the Office of Human Research Participant Protection (#10651). Diseased sites were defined as having evidence of clinical attachment loss, probing depth greater than or equal to 5 mm, and bleeding on probing. Healthy sites were defined as those that had a probing depth of 3 mm or less and no bleeding on probing. Subgingival plaque samples were taken using the method of Socransky *et al*. [36]. Briefly, after removal of supragingival plaque, subgingival plaque samples were collected with separate individual sterile Gracey curettes from the deepest site of each tooth. The samples were placed in separate microfuge tubes containing phosphate buffered saline (PBS) and then immediately processed for downstream analyses.

### Fluorescence *in situ* hybridization

Subgingival plaque samples were first fluorescently labeled for surface sialic acid using the labeling procedure described previously. Afterward, the samples were analyzed by fluorescence *in situ* hybridization (FISH). The protocol for FISH analysis of subgingival plaque samples was performed using a previously described methodology [37] and is briefly summarized. Plaque samples were fixed by suspending in 4% (vol/vol) paraformaldehyde at 4 °C for 1 hr and then repeated. Fixed samples were dehydrated in 50% (vol/vol) ethanol and stored at −20 °C for later use. Samples were diluted 1∶2 with 50% (vol/vol) ethanol and spotted onto a microscope slide in 10 µl total volume. Samples were allowed to air dry before adding 8 µl of hybridization solution [0.9 M NaCl, 20 mM pH 8 Tris-HCl, 35% (vol/vol) formamide, and 0.01% (wt/vol) SDS] and 1 µl (50 ng) of Alexa488 labeled FUS644 probe [38]. Samples were hybridized for 2.5 hr at 46 °C. After hybridization, samples were briefly rinsed with washing buffer [20 mM pH 8 Tris-HCl, 70 mM NaCl, 5 mM EDTA, 0.01% (wt/vol) SDS] preheated to 48 °C and then submerged in washing buffer for 15 min. at 48 °C. Samples were then rinsed with ddH_2_O and allowed to air dry before imaging.

### Fluorescent detection of sialidase activity

Freshly collected subgingival plaque samples were briefly sonicated to disperse cells and then resuspended in phosphate buffered saline (PBS) to a final OD_600_ ∼1.0. 10 µl of each cell suspension was then pipetted onto a glass microscope slide in duplicate. The protocol for sialidase detection has been previously described [39] and is briefly summarized. One set of test samples received 0.8 µl of 0.1 mM trifluoromethylumbelliferyl-α-D-N-acetylneuraminic acid (CF_3_MU-Neu5Ac) (Toronto Research Chemicals), while the other received the same volume of water. Samples were allowed to stand for an additional 3 min. at room temperature before directly observing fluorescence in the presence of UVA irradiation (365 nm).

## Results

### Exposure to whole blood activates expression of a sialic acid catabolism locus

Unlike many other species in the oral biofilm, *F. nucleatum* is a common constituent of both healthy and diseased subgingival sites [18], which suggests that this species is adept at growth in two vastly different environments. Given that extremely little is known about gene regulation in all oral fusobacteria, we were curious to examine the impact of whole blood upon the *F. nucleatum* transcriptome, since the presence of blood cells and serum components would be one of the major environmental distinctions between health and disease. Thus, as described in Materials and Methods, we grew *F. nucleatum* ssp. *nucleatum* ATCC 25586 in a semi-defined medium (SDM) to early log phase and then added defibrinated whole blood at a final concentration of 33% (vol/vol) to half of the culture and incubated for an additional 3 hr before extracting RNA. Using a 2-fold cutoff value, we identified >75 genes that were affected by the presence of added blood. Of these, only 25 genes exhibited increased expression, whereas twice as many genes had reduced expression. The largest gene expression changes occurred in the FN0663 – FN0664 operon and with FN1893. Both exhibited ∼10-fold lower expression in the presence of blood (Table 3). Interestingly, we also observed increased expression in a putative operon containing multiple genes with predicted roles in sialic acid catabolism (FN1470 – FN1476) (Table 3). As a final verification of the microarray data, we repeated the blood experiment twice and used qRT-PCR to assay the transcript levels of a variety of genes identified from the microarray dataset. As shown in Table 3, all of the trends in gene expression were identical between the qRT-PCR and microarray experiments. Though, the expression changes for several of the genes, such as FN1472 appeared to be underestimated by microarray.

**Table 3:** Selected genes from the microarray dataset and qRT-PCR confirmation*.

Locus Tag	Name/Putative function	Microarray	qRT-PCR	
FN0114	*grpE*; heat shock protein cofactor	0.27	0.43	0.24
FN0664	*fabK*; 2-nitropropane dioxygenase	0.12	0.02	0.06
FN1273	*tolC*; outermembrane efflux protein	0.27	0.26	0.24
FN1472	N-acetylneuraminate-binding protein	2.1	5.42	21.8
FN1893	Autotransporter outermembrane protein	0.09	0.01	0.03
FN1941	*clpB*; Clp protease ATP-binding subunit	0.27	0.35	0.2

*Only one gene per operon is listed. The complete microarray dataset is deposited in the GEO database under the accession number GSE36410.

### Examination of the *nan* operon

**Figure 1:**
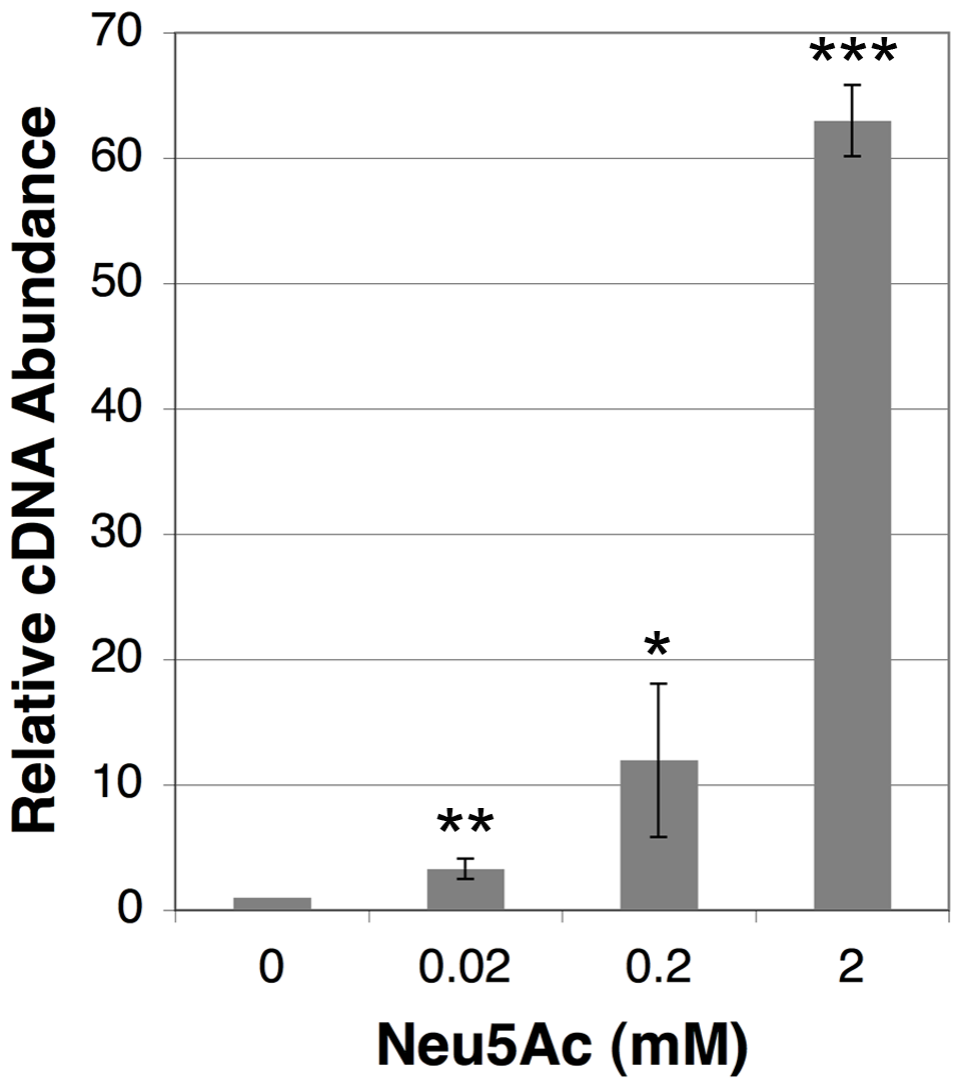
Effect of synthetic sialic acid upon *nan* operon transcription. *F. nucleatum* ssp. *nucleatum* was incubated in the presence of synthetic sialic acid at concentrations ranging from 0.02 mM to 2 mM before extracting RNA and measuring *nan* operon expression. Data are expressed relative to the cDNA abundance measured in the sample receiving no exogenous sialic acid. This sample was arbitrarily assigned a value of 1. All data were normalized using the DNA gyrase gene *gyrA* as a housekeeping control. Presented data are the average results from 3 independent experiments. Statistical significance was evaluated using an unpaired Student's *t*-test. *p<0.05, **p<0.01, ***p<0.001.

From the microarray and qRT-PCR experiments, it was clear that blood could serve as a strong inducer of the putative sialic acid catabolism operon FN1470 – FN1476 (Table 3). However, this result was also somewhat perplexing for a couple reasons. Firstly, fusobacteria are known amino acid fermenting, asaccharolytic organisms that are not known to use sugars as major sources of ATP [18]. Secondly, the concentration of free sialic acid in fresh serum is quite low [40], [41] and sialidase homologs are notably missing from the *F. nucleatum* genome. Thus, blood was not expected to be a rich source of metabolizable sialic acid in our assay. In addition, the growth medium already contained an excess of the preferred carbon source (peptides), which made nutrient limitation an even less likely explanation. Given these apparent inconsistencies, we were curious to examine the locus further. The FN1470 – FN1476 operon encodes all of the necessary functions expected from a typical *nan* operon: a specific TRAP transporter (FN1472 and FN1473), the core catabolic enzymes NanA, K, and E (FN1474– FN1476), an operon transcription regulator (FN1471), and even a sialic acid mutarotase NanM (FN1470) to facilitate the transport of exogenous sialic acid [3], [42]. Assuming the operon truly functions in sialic acid catabolism, we anticipated that it would be inducible using pure sialic acid (Neu5Ac). Since sialic acid in serum ranges from about 20 µM (free sialic acid) to 2 mM (total sialic acid) [40], [41], we assayed the expression of the putative *nan* operon within this range of exogenously added sialic acid. As shown in figure 1, *nan* operon transcription was inducible at sialic acid concentrations even as low as 20 µM and increased in expression proportionally with sialic acid concentrations up to 2 mM. From these data, it seemed highly likely that the *nan* operon indeed functioned in sialic acid catabolism. The next question then was whether sialic acid could be used as a carbon source like the many other species that encode similar *nan* loci [3]. Surprisingly, we found no evidence that sialic acid could substitute for peptides as a carbon source in SDM, as peptides seemed to be an obligate requirement for growth (data not shown). In fact, when 20 mM sialic acid was added to SDM containing a peptide carbon source, this actually resulted in substantially *slower* growth (data not shown), indicating some degree of toxicity.

### *F. nucleatum* ssp. *nucleatum* actively sialylates its cell surface

**Figure 2:**
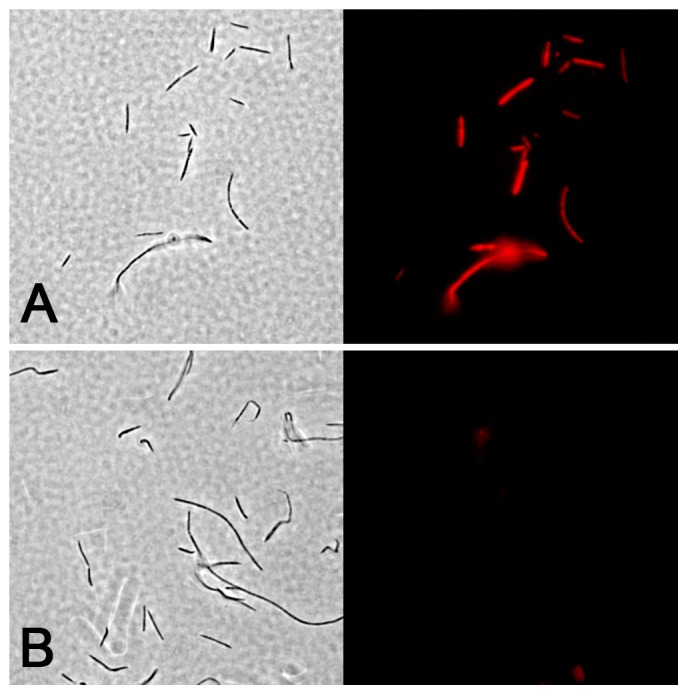
*F. nucleatum* ssp. *nucleatum* actively sialylates its outer surface. A) *F. nucleatum* ssp. *nucleatum* was grown in SDM before fluorescently labeling surface sialic acids. B) *F. nucleatum* ssp. *nucleatum* was grown in the presence of SDM and pretreated with neuraminidase before fluorescently labeling surface sialic acids. All pictures in the left panels are phase contrast images, whereas the right panel images are the corresponding images captured using epifluorescence microscopy. Total magnification is 1000x.

Even though the *nan* operon was responsive to the presence of sialic acid, our results suggested that this molecule was highly unlikely to play any prominent role in energy generation. For this reason, we suspected that sialic acid uptake might function in a different capacity. Certain bacteria are known to use sialic acid to decorate their outer surfaces, presumably as a mechanism to modulate innate immunity mechanisms. Therefore, we were curious to examine whether *F. nucleatum* might possess a similar cell surface sialylation ability. In order to detect this, we adapted a mild periodate oxidation labeling protocol described by Zeng *et al.* [35] to fluorescently label surface sialic acids. We initially grew *F. nucleatum* both in the presence and absence of blood to determine whether blood exposure would trigger surface sialylation. From these experiments, the specific effect of blood was difficult to ascertain, since *F. nucleatum* exhibits a potent blood agglutination activity that resulted in large conglomerates of fluorescently labeled blood cells intermixed with bacteria (data not shown). However, we were surprised to discover that fluorescent bacteria were easily detectable in the blood-free cultures (Fig. 2A). To determine whether the labeling was indeed specific to sialic acid, we repeated the experiment, but pretreated the cells with *Clostridium perfringens* neuraminidase before sialic acid labeling. As expected, neuraminidase treated cells were completely devoid of fluorescence (Fig. 2B) suggesting that the surface sialic acid had been digested from the cells. Consistent with its sialylation phenotype, were also able to identify the FN1682 – FN1686 operon as a likely *neu* operon utilized for *de novo* sialic acid synthesis. This operon is situated in a chromosomal locus containing many genes with predicted roles in lipopolysaccharide (LPS) synthesis/modification and the operon itself contains a likely *neuB* ortholog (FN1684) as well as a gene encoding a protein with both NeuA and NeuB domains (FN1686) along with at least two other genes with predicted roles in LPS or cell surface modification (Table 4). Interestingly, there is no obvious candidate for *neuC* in this operon, which indicates that *de novo* sialic acid synthesis would either require an alternative source of N-acetylmannosamine (ManNAc) or UDP-N-acetylglucosamine 2-epimerase activity is provided by an enzyme with little homology to known NeuC orthologs. Regardless, *F. nucleatum* ssp. *nucleatum* is apparently capable of both sialic acid catabolism and synthesis.

**Table 4:** Representative *neu* genes of sequenced oral fusobacteria.

Locus Tag	Pfam description/BLASTP match	Putative function
ssp. *nucleatum*		
FN1683	Acetyltransferase 4 family; GNAT domain	Neu5Ac modification?
FN1684	NeuB family; SAF domain (NeuB)	Neu5Ac synthesis
FN1685	dTDP-4-dehydrorhamnose reductase	LPS/cell surface modification
FN1686	NeuB and CTP transferase 3 family (NeuB/NeuA)	CMP-Neu5Ac synthesis
ssp. *polymorphum*		
FNP_1104	UDP-N-acetylglucosamine 2-epimerase (NeuC)	ManNAc synthesis
FNP_1105	CTP transferase 3 and Lipase DGSL2 family (NeuA)	CMP-Neu5Ac synthesis
FNP_1106	NeuB family; SAF domain (NeuB)	Neu5Ac synthesis
FNP_1107	Bacterial transferase hexapeptide; sialic acid O-acetyltransferase (NeuD)	Neu5Ac synthesis/modification
ssp. *vincentii* *		
FNV1764	UDP-N-acetylglucosamine 2-epimerase (NeuC)	ManNAc synthesis
FNV1765	CTP transferase 3 and Lipase DGSL2 family (NeuA)	CMP-Neu5Ac synthesis
FNV1766	NeuB family; SAF domain (NeuB)	Neu5Ac synthesis
FNV1767	NeuB family; Bacterial transferase hexapeptide; sialic acid O-acetyltransferase (NeuB/D)	Neu5Ac synthesis/modification
FNV1768	CTP transferase 3 (NeuA)	CMP-Neu5Ac synthesis
*F. periodonticum*		
FPOG_00225	NeuB family; Bacterial transferase hexapeptide; sialic acid O-acetyltransferase (NeuB/D)	Neu5Ac synthesis/modification
FPOG_00226	CTP transferase 3 and Lipase DGSL2 family (NeuA)	CMP-Neu5Ac synthesis
FPOG_00227	UDP-N-acetylglucosamine 2-epimerase (NeuC)	ManNAc synthesis

*F. nucleatum ssp. *vincentii* and ssp. *fusiforme* have been proposed to comprise a single subspecies [33].

### Sialic acid catabolism varies among oral fusobacteria, whereas surface sialylation is conserved

We were next curious to determine which aspects of sialometabolism may be shared among the other sequenced fusobacteria. Consistent with a previous report, we were unable to identify anything that resembled a *nan* operon in *F. nucleatum* ssp. *polymorphum* ATCC strain 10953 [17] or in strain F0401. However, using sequence data obtained the NCBI and HOMD databases together with Pfam analyses, we were able to identify a likely *nan* operon in ssp. *vincentii* (FNV1695 – FNV1703) that was identical in arrangement to that of ssp. *nucleatum* and even exhibited 96% nucleotide sequence identity over the entire length of the operon. The same was true of *F. nucleatum* ssp. *animalis* strains 11_3_2 (HMPREF0401_01326 – HMPREF0401_01332) and D11 (PSAG_01008 – PSAG_01014), but not strain 4_8. This suggests that both the *vincentii* and *animalis* subspecies are highly likely to catabolize sialic acid in a strain-specific manner, whereas this probably does not occur in the *polymorphum* subspecies. Likewise, no obvious *nan* operons were present in the three sequenced *F. periodonticum* strains D10, 2_1_31, or 1_1_41FAA. In contrast, sialic acid synthesis operons appear to be widely conserved among oral fusobacteria. *F. nucleatum* ssp. *nucleatum, polymorphum,* and *vincentii* as well as *F. periodonticum* were all found to contain unique operons encoding most or all of the necessary enzymatic activities required to synthesize sialic acid *de novo* (Table 4). In addition, each of the putative *neuA, B,* and *C* genes are located within larger LPS synthesis/modification operons/loci on the chromosome. Unlike the *nucleatum* subspecies, obvious *neuC* orthologs exist in both ssp. *polymorphum* (FNP_1104) and ssp. *vincentii* (FNV1764) and in *F. periodonticum* (FPOG_00227), which suggests that each possess complete *de novo* sialic acid synthesis capabilities. These three organisms also encode putative *neuD* sialic acid O-acetyltransferases (FNP_1107, FNV1767, and FPOG_00225) that are likely involved in adding O-acetylation to the sialic acid molecules [43], [44]. This activity appears to be missing from the ssp. *nucleatum neu* operon. However, its *neu* operon does encode a putative acetyltransferase (FN1683) of another variety, which may indicate that ssp. *nucleatum* utilizes sialic acid acetylation as well. Previous studies have shown that O-acetyltransferases are a common component of *neu* operons and play an important role in modulating the role of sialylation in host-pathogen interactions [43], [45]. Interestingly, we could not identify obvious orthologs of *neuA, B,* and *C* in the *F. nucleatum* ssp. *animalis* genome, but did identify a potential *neuD* ortholog (HMPREF0409_01489).

### Comparison of surface sialylation among oral fusobacteria

**Figure 3:**
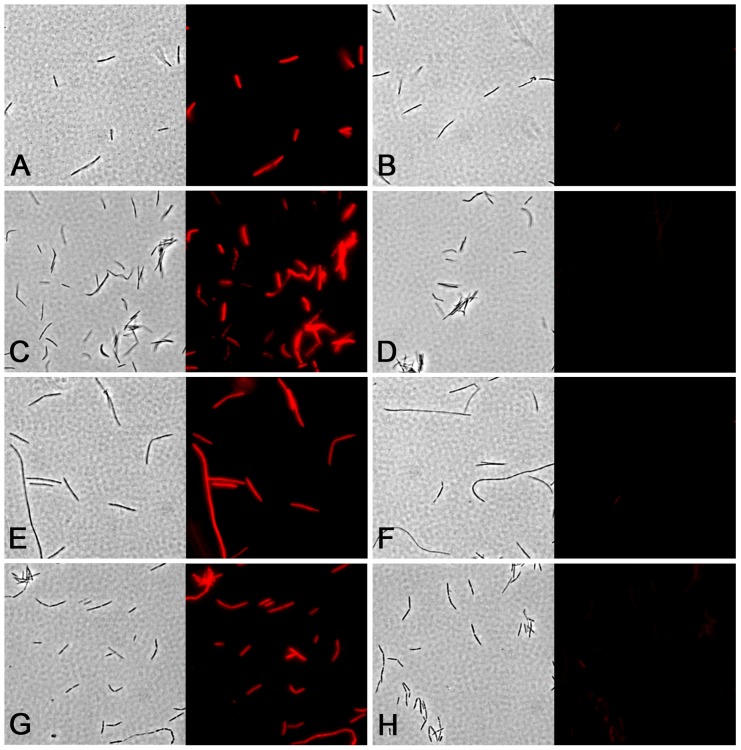
Surface sialylation is conserved among oral fusobacteria. Fusobacterial clinical isolates were grown in SDM to mid-log phase before fluorescently labeling surface sialic acids. Each set of images is shown with the phase contrast image on the left and the corresponding epifluorescence image on the right. The pictured species are: A) *F. nucleatum* ssp. *animalis*, B) *F. nucleatum* ssp. *animalis* pretreated with neuraminidase, C) *F. nucleatum* ssp. *vincentii*, D) *F. nucleatum* ssp. *vincentii* pretreated with neuraminidase, E) *F. nucleatum* ssp. *polymorphum*, F) *F. nucleatum* ssp. *polymorphum* pretreated with neuraminidase, G) *F. periodonticum,* and H) *F. periodonticum* pretreated with neuraminidase. Images were captured at 1000x total magnification.

**Figure 4:**
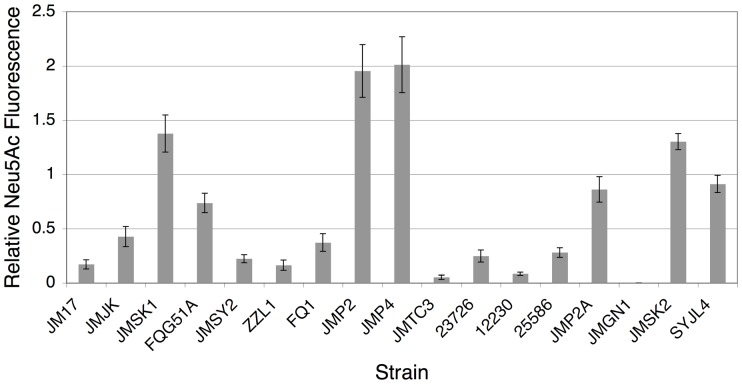
Comparison of surface sialylation abilities among oral fusobacteria. Representatives of all of the *F. nucleatum* subspecies as well as *F. periodonticum* were grown to mid-log phase in SDM containing vegetable peptone before fluorescently labeling surface sialic acid. Average fluorescence intensity values were determined using ImageJ image analysis software. Data are expressed relative to the average fluorescence intensity values for erythrocytes labeled in parallel. Erythrocyte fluorescence values were arbitrarily assigned a value of 1. The species identities for the strains are as follows: *F. nucleatum* ssp. *animalis* (JM17 – FQG51A), *F. nucleatum* ssp. *polymorphum* (JMSY2 – JMTC3), *F. nucleatum* ssp. *nucleatum* (23726 – 25586), *F. nucleatum* ssp. *vincentii* (JMP2A), *F. nucleatum* unnamed subspecies (JMGN1), and *F. periodonticum* (JMSK2 and SYJL4).

Since genomic analyses suggested that sialic acid synthesis was widely conserved among fusobacteria, we were interested to compare their surface sialylation characteristics. For this, we chose clinical isolates of *F. nucleatum* ssp. *animalis, vincentii,* and *polymorphum* as well as *F. periodonticum*. As shown in figure 3, each of these isolates exhibited neuraminidase sensitive fluorescent labeling of surface sialic acid, which suggested that sialylation was likely to be a general ability of oral fusobacteria even in *F. nucleatum* ssp. *animalis*. We also observed that some strains yielded greater fluorescence than others implicating variable levels of surface sialylation between strains. To explore the range of *de novo* sialic acid synthesis and surface sialylation potential among oral fusobacteria, we compared the density of sialic acid labeling in a panel of strains relative to that of mammalian erythrocytes. To exclude the possibility that sialic acid was scavenged from medium components, we replaced the animal peptone in the SDM recipe with that of vegetable peptone, since plant proteins contain little to no detectable sialic acid [46]. All other medium components were chemically defined and presumably free of sialic acid. Of the 17 strains tested, all but 1 exhibited easily detectable surface sialylation (Fig. 4). Surprisingly, two of the *F. nucleatum* ssp. *animalis* strains JMSK1 and FQG51A exhibited dense surface sialylation comparable to that of erythrocytes, whereas two *polymorphum* subspecies isolates yielded double the staining intensity of erythrocytes. Thus, there is an extremely wide range of sialylation potential among oral fusobacteria ranging from nearly undetectable sialylation (JMGN1) to even denser sialylation than mammalian erythrocytes (JMP4). This result is consistent with the extreme genotypic heterogeneity that exists among fusobacterial *neu* operons. It should also be noted that strain JMGN1 was our lone representative of an unnamed *F. nucleatum* subspecies identified by Kim *et al.* [33] and the only strain exhibiting almost no fluorescence. Therefore, it is still unclear whether other strains of this *F. nucleatum* subspecies actively sialylate or perhaps they require different growth conditions to trigger surface sialylation. Furthermore, for several strains, we observed that sialylation was not uniform among the population and only a percentage of the cells could be labeled (Table 5). However, most strains exhibited fluorescence in nearly 100% of the cells.

**Table 5:** Proportion of cells with detectable surface sialylation.

*Fusobacterium* species/subspecies	Strain	% Fluorescent cells
*F. nucleatum* ssp. *animalis*	JM17	100
	JMJK	60
	JMSK1	100
	FQG51A	100
*F. nucleatum* ssp. *polymorphum*	JMSY2	100
	ZZL1	55
	FQ1	100
	JMP2	95
	JMP4	100
	JMTC3	30
*F. nucleatum* ssp. *nucleatum*	ATCC 25586	95
	ATCC 23726	80
	ATCC 12230	95
*F. nucleatum* ssp. *vincentii* *	JMP2A	100
*F. nucleatum* (unnamed subspecies)	JMGN1	5
*F. periodonticum*	JMSK2	100
	SYJL4	100

*F. nucleatum ssp. *vincentii* and ssp. *fusiforme* have been proposed to comprise a single subspecies [33].

### Examination of fusobacterial sialylation characteristics within dental plaque

**Figure 5:**
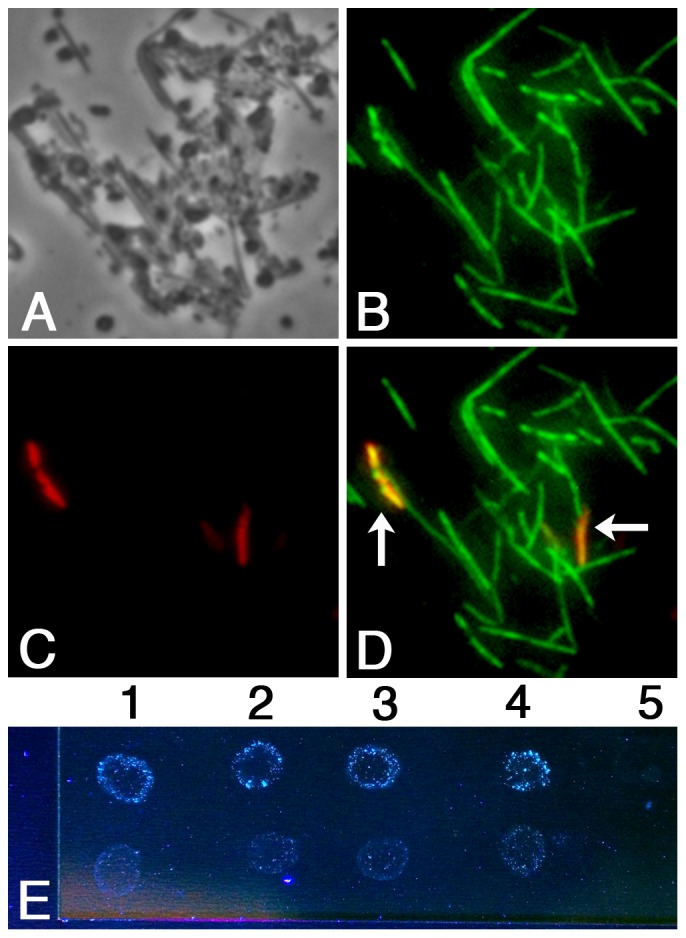
Analysis of fusobacterial surface sialylation in subgingival plaque. (A – D) Subgingival plaque samples were collected from both healthy and diseased sites and analyzed using a combination of fluorescence *in situ* hybridization (FISH) and fluorescent labeling of surface sialic acid. The health status of the patient sample had no discernable impact upon fusobacterial sialylation results and one representative sample is shown: A) phase contrast image of dispersed subgingival plaque, B) FISH results of subgingival plaque labeled with Alexa488-conjugated *Fusobacterium* genus probe FUS644, C) fluorescent image of sialylated bacteria in subgingival plaque, and D) merged image of FISH and sialylation images (arrows indicate doubly labeled cells). E) In rows 1–4, individual subgingival plaque samples were suspended in phosphate buffered saline (PBS), dispersed, and directly spotted onto a microscope slide in duplicate. Samples in the top row received 4-trifluoromethylumbelliferyl-α-D-N-acetylneuraminic acid (CF_3_MU-Neu5Ac), while samples in the bottom row received the same volume of ddH_2_O. In row 5, PBS containing CF_3_MU-Neu5Ac was spotted as a negative control. Samples were left at 25 °C for 3 min. before directly visualizing in the presence of UVA irradiation (356 nm). Visible CF_3_MU-Neu5Ac fluorescence is indicative of sialidase activity [39].

Given the high prevalence of surface sialylation among cultures of oral fusobacteria, we were curious whether cells in dental plaque would be similarly sialylated. To test this, we collected a total of 20 subgingival plaque samples: 10 from periodontally healthy sites and 10 from diseased sites. The samples were first stained for surface sialic acid to label sialylated bacteria and then analyzed by fluorescence *in situ* hybridization (FISH) to identify fusobacteria. As a control, we also confirmed that the double labeling approach yielded the expected sialylation and FISH results from a variety of the previously tested *F. nucleatum* and *F. periodonticum* isolates (data not shown). Interestingly, for both the health and disease plaque samples, we only observed a small minority of fusobacterial cells exhibiting detectable sialylation, even though numerous fusobacteria were identified in all samples (Fig 5A – D). We also cultured the plaque samples in SDM to determine whether the low frequency of sialylation was as a result of low viability among the fusobacteria in the clinical samples. However, we observed the same phenomenon. Despite extremely high numbers of fusobacteria, cultured plaques had few detectably sialylated cells (data not shown), unlike the pure cultures of our clinical isolates grown in the same medium (Table 5). This implicated the multispecies environment as somehow responsible. Given that we had already confirmed the neuraminidase sensitivity of fusobacterial sialylation, we were curious whether sialidase activity in dental plaque [17] could be a contributing factor. Thus, we added the fluorescent sialidase substrate trifluoromethylumbelliferyl-α-D-N-acetylneuraminic acid (CF_3_MU-Neu5Ac) to plaque samples taken from both healthy and diseased sites to assay total sialidase activity. Surprisingly, even small volumes of diluted plaque from either source contained enough sialidase activity to directly visualize CF_3_MU-Neu5Ac fluorescence (Fig. 5E). The usual fluorometer measurements were unnecessary for detection. This indicated that sialidase activity in dental plaque is extraordinarily high irrespective of health status and suggested a potential role for sialidases in removing terminal sialic acid residues from sialylated fusobacteria.

## Discussion

In the current study, we utilized microarray analysis to characterize the response of *F. nucleatum* ssp. *nucleatum* to whole blood. As a result of these studies, we identified two independent metabolic pathways for sialic acid catabolism and synthesis. The capacity for sialic acid synthesis and/or surface sialylation was determined to be a highly conserved ability among oral fusobacteria, whereas sialic acid catabolism is likely to be subspecies- or strain-specific. Interestingly, our results suggest that fusobacterial strains capable of sialic acid catabolism are unlikely to use this molecule for nutrition to the same extent seen in other sialic acid catabolizing species.

Overall, the microarray results indicate that blood exposure represses many more *F. nucleatum* pathways than it activates. Many of the genes with lower expression are important components of the stress response machinery, such as *grpE* (FN0114), *clpB* (FN1941), and others. Such a result seems reasonable, since fusobacteria typically grow much better in media that contain blood. Of particular note, blood caused a potent reduction in expression (33–100-fold) from an uncharacterized gene (FN1893) encoding a large outermembrane autotransporter protein (>2300 residues) (Table 3). FN1893 is highly conserved among all subspecies of *F. nucleatum* and in *F. periodonticum* as well as in members of the closely related genus *Leptotrichia*. *F. nucleatum* encodes a variety of analogous large outer membrane autotransporter proteins and all characterized examples function as bacterial and/or host cell adhesins [47]–[49]. Therefore, it is intriguing that the presence of blood would have such a dramatic effect upon this particular gene and not the other autotransporter adhesin-like genes in the genome. Of the relatively few genes induced by the presence of blood, it was particularly surprising to observe induction of a putative *nan* operon, since it was expected that the growth medium contained a very low concentration of free sialic acid. Also, *F. nucleatum* possesses a limited capacity for sugar catabolism in general [18].

Recent *in silico* analyses of *F. nucleatum* have predicted that both sialic acid catabolism [3], [50] and cell surface sialylation [17], [50] should occur among the various *F. nucleatum* subspecies. Though, neither abilities had been demonstrated experimentally. Despite the presence of a sialic acid-inducible *nan* operon in *F. nucleatum* ssp. *nucleatum,* we found no evidence that it could utilize sialic acid as a major energy source. In fact, the apparent toxicity we observed when ssp. *nucleatum* was grown in 20 mM sialic acid likely attests to its limited catabolic capacity. If sialic acid were transported into the cell at a rate greater than its ability for catalysis, we would anticipate a growth inhibitory effect. Currently, it is unclear what advantage the *nan* operon may impart to the *F. nucleatum* strains encoding this operon, but one possibility is that the high affinity TRAP transporter in the operon may provide a source of exogenous sialic acid that could be used for surface sialylation. The remaining catabolic enzymes encoded in the *nan* operon may serve to prevent toxicity arising from an intracellular accumulation of the transported sialic acid. Our data also suggest that the limited quantity of free sialic acid in serum may be sufficient to induce *nan* operon expression (Table 3 and Fig. 1). Additionally, the extremely high sialidase activity present in dental plaque (Fig. 5E) might further increase the local concentration of freely available sialic acid, particularly in the presence of saliva or serum. Interestingly, the capacity for sialic acid catabolism is not nearly as widely conserved among oral fusobacteria as sialic acid synthesis. Nearly every isolate from a wide range of oral fusobacteria exhibited substantial surface sialylation on most or all cells when grown in a medium containing no exogenous source of sialic acid (Table 5). Similarly, potential *neu* gene operons could be identified in the genomes of most sequenced oral fusobacteria with *F. nucleatum* ssp. *animalis* being the lone exception. Currently, it is unclear which genes are responsible for sialic acid synthesis in this subspecies.

It was quite surprising to discover such a large discrepancy between the sialylation state of fusobacteria in pure culture vs. in dental plaque. In the oral cavity, the vast majority of fusobacteria are found within polymicrobial biofilms, and yet our results indicate that very few cells exhibit detectable sialylation in a multispecies environment. Despite this, nearly every *F. nucleatum* and *F. periodonticum* isolate constitutively produced some degree of surface sialylation in pure culture with numerous strains exhibiting similar or greater sialylation densities compared to mammalian cells. For this reason, we predict that fusobacterial surface sialylation in dental plaque is likely to be more prevalent than the staining results indicate. We suspect this to be as a consequence of sialidase activity in dental plaque. Since fusobacteria could be desialylated with neuraminidase (Figs. 2 & 3), the potent sialidase activity we detected in dental plaque (Fig. 5E) would be similarly expected to continually digest terminal sialic acids from fusobacteria. In this case, few fusobacterial cells should exhibit surface sialylation. While it may appear to be a futile cycle of sialic acid synthesis and digestion by sialidases, the continual removal of sialic acids from fusobacteria may hint at an interesting niche for these organisms in polymicrobial biofilms of the mucosa. By synthesizing sialic acid *de novo*, fusobacteria may be providing an important alternative source of sialic acid for the many sialic acid catabolizing species located in the interior of the biofilm. Presumably, it is the surface layers of the biofilm that encounter the highest concentration of free sialic acids liberated from mucosal glycoproteins, whereas regions further inside the community are likely depleted of this energy source. Metabolic complementation by fusobacteria may be even further enhanced by the strong coadherence between fusobacteria and the numerous sialidase producing species in the biofilm. Furthermore, very few bacterial species have been reported to synthesize sialic acid *de novo*, which could make fusobacteria particularly important for supporting the long-term persistence of the various mucosal communities where these species are found. Unfortunately, the genomic heterogeneity of *neu* loci in oral fusobacteria (Table 4) complicates *in situ* examinations of *neu* gene expression in clinical plaque samples. Thus, we currently cannot exclude the possibility that a multispecies environment simply serves as a repressor of *neu* gene expression. A defined *in vitro* assay system would likely be required to reconcile this possibility. Furthermore, since oral fusobacteria exhibit tenacious adherence to numerous human cell types, it will be interesting to determine whether strain-specific sialylation characteristics also play a role in modulating the host inflammatory response. Several sialylated mucosal pathogens have been previously demonstrated to alter human immune cell signaling pathways by directly engaging siglec receptors [12], [13], [15], [51]. It is conceivable that fusobacteria could play a similar role outside of the biofilm environment.
